# Autism Spectrum Disorder in adults: an integrative review about strategies for promotion and maintenance of quality of life

**DOI:** 10.1590/1516-3180.2024.0324.R1.14042025

**Published:** 2025-07-11

**Authors:** Gabriela Garcia de Carvalho Laguna, Isadora Bagues Rodrigues, Sara Emanuelle dos Santos Neves, David Santos Libarino, Fernanda Beatriz Melo Maciel, Antônio Gonçalves Pessoa, Leticia Defensor da Silva Santos, Nilia Maria de Brito Lima Prado

**Affiliations:** I Instituto Multidisciplinar em Saúde, Universidade Federal da Bahia (UFBA), Vitória da Conquista (BA), Brazil.; II Instituto Multidisciplinar em Saúde, Universidade Federal da Bahia (UFBA), Vitória da Conquista (BA), Brazil.; III Instituto Multidisciplinar em Saúde, Universidade Federal da Bahia (UFBA), Vitória da Conquista (BA), Brazil.; IV Instituto Multidisciplinar em Saúde, Universidade Federal da Bahia (UFBA), Vitória da Conquista (BA), Brazil.; V Instituto Multidisciplinar em Saúde, Universidade Federal da Bahia (UFBA), Vitória da Conquista (BA), Brazil.; VI Instituto Multidisciplinar em Saúde, Universidade Federal da Bahia (UFBA), Vitória da Conquista (BA), Brazil.; VII Instituto Multidisciplinar em Saúde, Universidade Federal da Bahia (UFBA), Vitória da Conquista (BA), Brazil.; VIII Instituto Multidisciplinar em Saúde, Universidade Federal da Bahia (UFBA), Vitória da Conquista (BA), Brazil.

**Keywords:** Autism Spectrum Disorder, Adult, Aging, Quality of life, Mental health

## Abstract

**BACKGROUND:**

People with Autism Spectrum Disorder (ASD) experience phases of life beyond childhood, which increase the difficulties inherent in each cycle. In contrast, the scientific literature lacks broad reviews of developmental strategies regarding quality of life, even though ASD encompasses changes in social, communicative, and behavioral skills.

**OBJECTIVE:**

This study aimed to identify strategies for promoting and maintaining quality of life among adults diagnosed with autism.

**DESIGN AND SETTING:**

This integrative review was conducted in Brazil.

**METHODS:**

This review searched the Scopus and Web of Science databases, from strategies that combined the descriptors ("Autism Spectrum Disorder"), ("Autism"), ("Aging"), and ("Quality of life"). Original studies with the full text available in English published between 2018 and 2023 were included, if they responded to the eligibility criteria.

**RESULTS:**

In total, 3,098 studies were identified, of which 44 were selected to compose the bibliographic sample of this review. The population sample included 184,653 participants diagnosed with ASD, aged on average 43.5 years old. The following were described for adults with autism: 1) cognitive aspects, 2) aspects related to suffering/mental illness, and 3) strategies to promote quality of life.

**CONCLUSION:**

This research contributes to basic clinical practice and promotes responsible care that attends to the health needs of people with autism throughout life. Early interventions in autistic adults and the availability of support throughout life are essential for maintaining cognitive health and quality of life.

## INTRODUCTION

 Autism spectrum disorder (ASD) is a neurodevelopmental disorder involving changes in social, communicative and behavioral skills. Diagnosis is supported by stereotypical and repetitive behaviors (e.g., *flapping*), restricted interests, difficulty interpreting social scenarios, reduced peer interactions, diminished shared emotions, limited visual contact and vocal variation, rigidity with rules, resistance to change, and preference for objects due to texture or smell.^1^

 Its prevalence ranges from 1% to 2% of the global population, with rates increasing in recent research.^1^ Since Kanner conducted the first study with children diagnosed with autistic affective contact disorder, this neurodevelopmental disorder has been read as a childhood disorder, with literature on this disorder in adolescents, adults and elderly adults being scarce.^1-4^ Despite early conceptions of autism as a childhood disorder, projections estimate 1,7 million autistic individuals over age 65 in the United Kingdom by 2030,^5^ and 50 thousand autistic people enter adulthood annually in the United States.^10,6^ This underscores the necessity of research addressing autism across all life stages to inform clinical care. 

 Although some reviews focus on transitions to adulthood or psychosocial aspects of aging in ASD, studies exploring the condition in later life remain scarce.^7,8^ Approximately 70% of individuals with autism have at least one comorbid mental disorder, such as anxiety, depression, or structural language disorders, and 40% have two or more comorbidities.^2^ Medical conditions such as epilepsy, sleep disorders, and constipation further increase health risks, emphasizing the importance of strategies to enhance quality of life in this population.^1,2^

 By 2023, only 2% of ASD studies had addressed adults and elderly people,^9^ limiting evidence-based strategies for these groups. Analysis of the repercussions of the pandemic on neurodivergent children’s lives, reveal negative aspects, such as behavioral changes, mood, hyperactivity and communication;^10-12^ on other age groups are important for a broader understanding of these impacts. Additionally, low-income countries, including Brazil, often lack basic autism statistics, where data on aging in ASD are minimal.^11,13^ This hinders the planning of care for adults and elderly adults with ASD and limits research outcomes. 

 In line with the association of ASD with multiple comorbidities, the increase in this population beyond childhood and its greater vulnerability, as well as the lack of broad reviews regarding paths to healthy development, justify and reiterate the need for this review. Therefore, this article aimed to complement the scientific literature on this subject by proposing and identifying strategies for promoting and maintaining quality of life among adults diagnosed with autism to support adequate care for the characteristics of this population. 

## METHODS

 This integrative review aimed to promote a synthesis of knowledge on the issue: "What strategies can be adopted to improve and maintain the quality of life of people with autism after childhood?" The research was conducted in six stages: 1) elaboration of the guiding question; 2) literature search; 3) data collection; 4) critical analysis of the included studies; 5) discussion of the results; and 6) presentation of a review integrative.^14^

 The Health Sciences Descriptors (DeCS) used—("Autism Spectrum Disorder"), ("Autism"), ("Aging") It is ("Quality of Life")–were combined with the Boolean operator AND and applied in the following strategies of searches: 1) ("Autism Spectrum Disorder") AND ("Aging"); 2) ("Autism") AND ("Aging"); 3) ("Autism Spectrum Disorder") AND ("Aging") AND ("Quality of life"); 4) ("Autism") AND ("Aging") AND ("Quality of life"). This research was conducted in August 2023 using the Scopus and Web of Science databases. 

 Original studies with full text available in English, published in the last five years (2018–2023) that responded partially or fully to the research question were included as eligibility criteria. A study partially answered the research question when it did not directly present strategies to improve quality of life but contributed to the understanding of the experience of ASD in adult life by discussing neurocognitive aspects and/or aspects related to suffering or mental illness. Duplicate articles were excluded if in another language; other textual genres, such as letters, comments, reports, summaries, and editorials; incomplete articles, such as protocols; reviews of any type; articles unavailable, considering a failed access attempt via the institution and a request to authors not responded to; in addition to studies not related to the objective of the research, that is, whose population was 1) people under 18 years of age and/or 2) people without a formal diagnosis of ASD, such as people with an extended phenotype and/or family members/caregivers; and/or 3) those that did not address issues related to growth, aging, and/or quality of life of adults with ASD. 

 Initially, studies were screened by reading the titles and abstracts, with the help of software Rayan,^15^ followed by reading the eligible articles in full. This stage was performed by two independent, blinded reviewers (GGCL and FBMM). The selected studies were systematized in a database using Microsoft Excel®, considering the following variables: authors, year of publication, study location, study design, sample (number of participants, age range and gender ratio when informed), main results, strategies to promote quality of life in adults with ASD, and limitations of the study. 

 Systematization involved the stages of identification, registration, analysis, and interpretation of the selected studies. Numerical data are presented based on the main results and strategies to promote quality of life in adults with ASD. Descriptive statistics, in absolute numbers and percentages, and qualitative data were categorized considering 1) cognitive aspects, 2) suffering/mental illness, and 3) strategies to promote quality of life. 

## RESULTS

**Figure 1:**
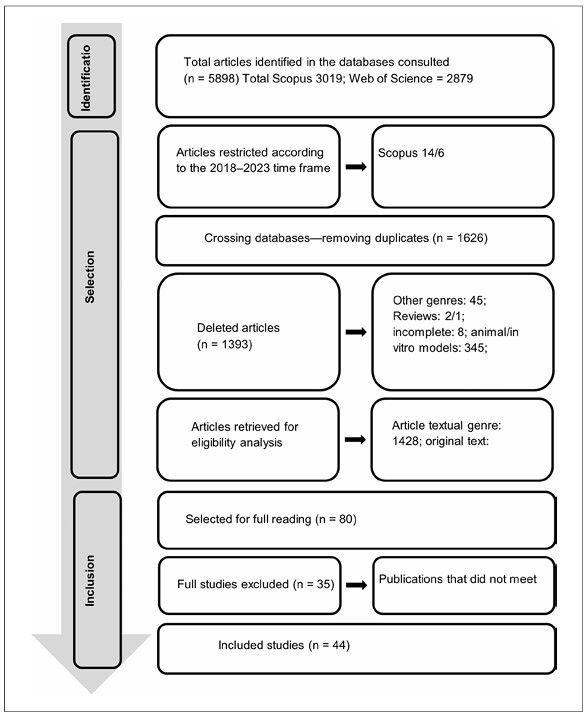
Flow chart bibliographic selection.

 In total, 3.098 studies were published between 2018 and 2023 (Scopus = 1.476, Web of Science = 1,622), of which 44 were selected to compose the bibliographic sample of this review according to the inclusion and exclusion criteria. **Figure 1** illustrates this process. 

### Characterization of studies

 The sample of 44 articles was composed of 651.738 participants, of whom 184,653 (28.33%) were diagnosed with ASD, with ages ranging from 18 to 92 years, with an average age of 43.5 years. Among the selected articles, 95,4% were mixed, comprising men and women, and one article was composed exclusively of men and two exclusively of women. 

 All analyzed articles were conducted in developed countries: the United States (50%), the Netherlands (13.63%), the United Kingdom (11.36%), France (6.81%), Australia (6.81%), Germany (4.54%), South Korea (2,27%), Canada (2,27%), England (2,27%), and countries of Europe unspecified (2,27%). The study designs were mainly observational (95.45%), with only two being interventional. The individual characteristics of the studies are summarized in **Table 1** . 

**Table 1:** Individual characterization of studies

**Author and year**	**Study design and location**	**Sample**	**Main results**	**Strategies to promote quality of life in adults with ASD**
Abbott, Happé and Charlton(2018)^19^	Cross-sectional study (UK)	134 participants diagnosed with ASD (97 men and 37 women). Age range: 18–75 years old. Average age: 31.14.	Adults diagnosed late with ASD showed better performance in certain cognitive skills, such as speed and sequencing than in typical age norms, while other executive skills followed normal aging patterns.	Include therapies that aim to improve executive functions, adequate physical activity, and specialized medical monitoring. Additionally, considering group activities, social practices, and interventions specific to comorbidities may be beneficial. However, approaches should essentially be personalized based on individual needs, such as promoting autonomy and reducing anxiety.
Abigail Dickinson et al.(2022)^25^	Cross-sectional study (USA)	180 participants (93 diagnosed with ASD and 87 controls without diagnoses of ASD). Age range: 18–70 years old.	Neurophysiological aging was shown to be accelerated in adults with ASD, compared to adults without ASD. However, nonverbal cognitive abilities in both groups are similar, which suggests that oscillatory characteristics associated with cognitive processing may vary.	Not described.
Baxter et al.(2019)^16^	Cross-sectional study (USA)	76 sex participants masculine(42 diagnosed with ASD and 34 controls with diagnosis of ASD). Age range: 18–60 years old.	The groups did not differ on estimated IQ and performance of fluency. Young and elderly adults with ASD showed poor performance on cognitive tests. Large age-related reductions in subcortical structures focusing on the left thalamus were observed in the study, which suggests possible weakening in frontal-subcortical connectivity in older adults or differences in processing internally directed task demands.	Using fluency networks helps us form an even clearer picture of how language is altered by aging. By a better understanding of the brain networks involved in producing similar levels of cognitive performance, it may also have implications for the ways in which these compensatory mechanisms may be involved in other cognitive areas.
Bathelt et al.(2020)^21^	Cross-sectional study (Netherlands)	51 participants diagnosed with ASD (68.6% men and 31.4% women). Age range: 30–74 years old. Average age of 45 years.	Aging has been shown to affect brain connectivity, especially reducing Default Mode connections. This indicates that aging and ASD affect the brain in specific ways.	Not described.
Bernhardt et al.(2020)^26^	Interventional study (USA)	5 participants diagnosed with ASD (3 men and 2 women). Age range: 20–25 years old.	A program was developed in which participants stayed in student residences, participating in activities: 1) in the house: house rules, safety plans, hygiene routines, household chores, meal preparation; and 2) on campus: participation in career development, credit and finance, first aid safety, arts, and local community workshops, with varying levels of support depending on the person, activity, and environment. The intervention proved to be effective, participants demonstrated the quality learning of life in all constructs of active engagements and expression of interest in future life projection.	Join some type of community where they can have support in reaching their own wellness goals.
Bishop-Fitzpatrick e Rubenstein(2019)^23^	Observational study (USA)	143 participants diagnosed with ASD. Age range: 40–88 years old.	Physical and mental health conditions such as immunological (70.6%), cardiovascular (49%), sleep disorders (85.3%), gastrointestinal (49.7%), neurological (55.9%) and psychiatric (72%) disorders were prevalent. Middle-aged and older adults with ASD and intellectual disability had higher prevalence of epilepsy and less of depression/anxiety compared to those without intellectual disabilities.	It highlights the importance of health professionals, especially those working in family health, internal medicine, and geriatrics, being aware of the multiple health conditions that can affect adults with ASD. This suggests the need for additional training for these professionals in treating chronic conditions associated with ASD.
Braden et al.(2022)^27^	Cross-sectional study (USA)	133 participants (67 diagnosed with ASD and 66 controls with no diagnosis of ASD). Age range: 18–71 years old.	A mindfulness-based intervention proved to be beneficial in improving quality of life related to psychological health and reducing difficulties related to disability in adults with ASD.	Support/education and stress reduction interventions based on mindfulness improve quality of life related to mental health, being more effective for women with ASD. Focusing on emotional awareness and attitudes of acceptance can be crucial to improving the quality of life of this population.
Bush et al.(2018)^28^	Cross-sectional study (Canada)	496 participants (248 diagnosed with ASD and 248 controls which diagnosis of ASD; all female participants). Age range: 18–30 years	Participants with ASD reported significantly lower sexual desire compared to participants without ASD, and less involvement in sexual behaviors throughout their lives, which included a significant reduction in the number of reported sexual activities. Sexual satisfaction does not differ significantly between the two sample groups. Notably, both had high rates of nonbinary or fluid gender identities, as well as a variety of sexual orientations.	Not described.
Chan et al.(2018)^2^	Longitudinal study section (USA)	406 participants (adolescents and adults diagnosed with ASD, 72.4% men). Age range: ≥ 18 years.	The most powerful predictors of whether a person with ASD and DID will remain in employment were living in an area with a larger population, having access to inclusive education as a child, and having more independent daily living skills. Family factors were also predictive, such as higher income and more extensive maternal social connections.	Individuals with ASD and DID deserve special attention in intervention and professional training programs related to daily living skills, prioritizing the teaching of self-care skills, training in domestic skills, and exposure to an inclusive lifelong learning environment.
Chan et al.(2023)^29^	Longitudinal study (USA)	40 Participants diagnosed with ASD (27 males and 13 females). Age range:≥ 18 years. Average age of 37 years.	Many adults with autism was shown to want social connections. The study provides initial support about the context where people with ASD seek to develop social interactions, namely: vocational contexts, neighborhoods, common interest groups, support services and inclusive environments, and networks and applications online.	Establishing in-person support groups with other adults with ASD who have common interests, for example: game nights, participation in religious communities, work, volunteering, online community participation specific to this population or creating an autism task force focused on supporting co-workers who are also on the spectrum.
Charlton et al.(2023)^30^	Cross-sectional study (USA)	388 participants diagnosed with ASD. Age range: 40–83 years.	Evidently, the subjective social support was positively associated with quality of life in older adults with autism. Symptoms of depression and anxiety had a negative impact on quality of life. Additionally, age positively affected the psychological and environmental quality of life, while sex assigned at birth influenced physical and environmental quality of life.	Positive social support impacts the quality of life in elderly and young adults with ASD. Subjective social support plays a significant role in all aspects of quality of life, while social interactions and instrumental support contribute to specific domains. Social support is crucial to quality of life in older adults with autism, considering demographic factors and depression.
Clark et al. (2023)^31^	Longitudinal study (USA)	151 participants diagnosed with ASD. Age range: 18–28 years old.	Notably, some particularities of the public with ASD that can influence their quality of life and professional stability: 1) importance of early interventions; 2) diversity in trajectories; and 3) access to services, that is, offering employment support.	Professional activities such as employment and postsecondary education increase subjective wellbeing in adults with ASD. Participants involved in independent vocational activities had higher ratings of wellbeing and a greater propensity for social contacts, highlighting the importance of these activities.
Crawley et al.(2020)^24^	Observational study (Europe)	321 participants diagnosed with ASD. Average age: 18–30 years.	The study revealed that adults with ASD show decreased mental flexibility, which is linked to repetitive behaviors and differences in the way they learn.	Understanding differences in behavior can guide personalized interventions, considering individual preferences, sensitivities, and learning patterns. Understanding how the learning environment influences performance can lead to more adapted and effective therapeutic approaches.
DaWalt et al.(2021)^22^	Observational study (USA)	2,187 participants diagnosed with ASD (78.54% men). Average age: 30.6 years.	The results suggest differences in the prevalence of diseases and use of healthcare related to ASD and gender, with women with ASD presenting greater risk and greater use of healthcare in various conditions. However, specific details about the medical conditions and use of health services related to ASD and gender are not described.	It suggests the intensification of the use of healthcare by women with ASD, considering biological differences, diagnostic processes, perceptions, and responses from society. Findings emphasize the importance of examining healthcare utilization alongside prevalence, highlighting the specific challenges faced by women with ASD, and advocating for personalized interventions and supports.
Gabbai and Garreau(2022)^32^	Cross-sectional study (France)	135 participants diagnosed with ASD (61.5% men and 38.5% women). Age range: 18–92 years old. Average age: 53 years old, with the majority of participants between 35 and 74 years old (85%).	The study highlights that in adulthood, the emphasis is on associated disorders and psychiatric comorbidities, instead of the fundamental characteristics for the diagnosis of ASD. It is important to adopt a functional approach that reveals the underlying psychological distress and the coping strategies developed by patients.	The relevance of clinically addressing associated disorders and psychiatric comorbidities, prioritizing a functional perspective that reveals patients’ adaptation to psychological distress. It concludes by highlighting the need for structured institutional work to deal with the diversity of these patients and ensure coherent approaches.
Geurts et al.(2020)^20^	Observational study (Netherlands)	101 participants diagnosed of ASD (all men and elderly adults). Average age: 60–85 years.	The results suggest that older men with autism diagnosed with ASD may report more challenges in cognitive flexibility, planning, processing speed, and working memory in daily life, although they do not show significant differences in performance on neuropsychological tests compared to older adults without ASD.	Strategies to improve the quality of life of these autistic adults may include interventions based on an understanding of these daily challenges, possibly involving training in internal or external strategies. Understanding the specific causes of these difficulties can guide personalized support.
Groenman et al.(2021)^33^	Longitudinal study (Netherlands)	135 female participants (58 diagnosed with ASD and 77 diagnosis with ASD). Age range: 31–73 years.	They were not identified statistically significant differences of the suffering of autistic women in relation to the disorder premenstrual dysfunction compared to non-autistic women. Autistic women presented higher psychological and somatic complaints, mainly ASD, also presenting complaints of ADHD, without an increase in urogenital complaints.	More substantial social support during stressful events, greater physical fitness, better coping strategies, and better sleep quality. Future studies are needed to analyze the link between menopausal complaints and estrogen levels, and the sensitivity to them shown by women with autism.
Hand et al.(2019)^34^	Case-control study (USA)	21,792 participants diagnosed with ASD. Age range: 18–59 years old.	Adults with autism were 4.3% in the study who had at least one medical encounter related to suicidal ideation, which is similar to estimated prevalence rates for the general population (3%-4%). However, 4.1% of adults with autism had at least one medical encounter related to suicide attempts or selfharm, which is significantly higher than the general population (0.4%-0.6%). Linked to this, risk factors such as bipolar and unipolar depression were strongly associated with suicidal ideation in people with ASD.	Adults with ASD and co-occurring intellectual disability had significantly lower odds of a medical encounter for suicidal ideation, but significantly higher odds of attempted suicide/self-inflicted injury.
Hau et al.(2021)^35^	Cross-sectional study (USA)	52 participants (28 diagnosed with ASD, 22 men and 6 women; and 26 controls without a diagnosis of ASD, 8 being men and 18 women). Age range: 40–70 years old.	Reduction in microstructural integrity of the left dorsal premotor tract (PMd) and supplementary motor area in each substrate (SMA-cst) was associated with greater severity of restrictive behavior in adulthood.	This finding may reflect a greater ability to appropriately select actions based on associated stimuli and inhibit behaviors, such as stereotypies, in individuals with more effective secondary motor relays.
Hwang, Foley e Trollor(2018)^36^	Cross-sectional study (Australia)	92 participants diagnosed with ASD. Age:≥ 40 years.	Evidently, the majority of adults with ASD do not meet the criteria for "successful aging" as defined by the Rowe and Kahn model, with difficulties in maintaining social relationships and engaging in activities of daily life, resulting in less social participation.	Notably, adults with autism face additional challenges, such as a greater number of medical comorbidities and difficulties accessing medical care, which requires greater attention to these specific demands. The need for strategies to promote social inclusion throughout the lives of these individuals is also highlighted.
Janice Hau et al.(2022)^37^	Cross-sectional study (USA)	73 participants (35 diagnosed with ASD and 37 controls which diagnosis of ASD). Age range: 41–70 years.	People with ASD have been shown to have a stronger connection between structural and functional brain connectivity, especially in the right hemisphere. Differences in the structure and connectivity of cerebral white matter exist in adults with ASD compared to neurotypical adults. However, these characteristics may vary depending on the activity that the adult with ASD is exposed to.	Not described.
Klein et al.(2022)^38^	Cohort study (USA)	210 participants diagnosed with ASD (89 males and 121 females). Average age: 55.63 years.	High rates (30%) of cognitive decline were found in autistic adults, with symptoms such as lack of interest in hobbies and memory problems. And that autistic women may be more vulnerable to decline, highlighting the importance of early screening and appropriate care.	Not described.
Lever e Geurts(2018)^39^	Cross-sectional study (Netherlands)	440 participants (241 with diagnosis of ASD and 199 controls without diagnosis of ASD).	Adults with ASD face difficulties related to empathy and sensory sensitivities throughout their lives, with characteristics more evident in middle age (49 years) and less in young/older adults.	Understanding the variability of differences in ASD characteristics across adulthood across the lifespan can guide personalized support approaches.
Link et al.(2021)^40^	Cross-sectional study (USA)	33 participants diagnosed with ASD. Average age: 40–65 years.	Adults with ASD have deficits in several motor skills, including manual dexterity, coordination, and strength and flexibility, compared to the control group. Furthermore, functional connectivity between sensorimotor regions was reduced in the ASD group, and connectivity patterns were more variable among individuals with ASD than in the control group.	Understanding variations in sensorimotor connectivity highlights the importance of personalized approaches and longitudinal monitoring to better understand changes over time. This may be crucial to develop specific interventions and improve the quality of life of adults with ASD, potentially preventing accelerated functional declines.
Mason et al.(2019)^41^	Cross-sectional study (UK)	69 Participants diagnosed with ASD (48 men and 21 women). Age range: ≥ 55 years.	Quality of life scores in all domains were lower for individuals who reached the points of clinical cutoff indicators for depression according to the Hospital Anxiety and Depression Scale (HADS) (*F* (8.126) = 6,171, P < 0.001); results were similar for anxiety (*F* (8.126) = 3,902, P < 0.001) with the exception of the social quality of life domain, where no significant differences were found in the score. Subjective quality of life did not differ according to participation in normative outcomes (*F* (12.124,64) = 1,363, P = 0,192).	Perceived informal rather than formal support predicted higher subjective quality of life. Religious communities may offer a consistent source of informal support and acceptance. Providing suitable environments for people with ASD is significantly related to subjective and focus on individual provision dualized.
Miot et al.(2019)^42^	Observational study (France)	63 participants diagnosed with ASD (3.7 men for each women). Average age: 43 ± 15.1 years.	The most frequent comorbidities identified were constipation (54%), epilepsy (28.6%), and chronic kidney disease (25.4%). Analyzes associated ASD severity with epilepsy. Comorbidities impacted communication skills and daily living. Comorbidity burden was influenced by age, polypharmacy, and level of autonomy.	Promoting autonomy and personalized assessment can improve the quality of life of adults with ASD and intellectual disabilities. Reducing polypharmacy and considering specific strategies for comorbidities such as constipation are important approaches. Close attention to inflammation and a comprehensive geriatric assessment may also benefit these individuals.
Miot et al.(2022)^43^	Observational study (France)	63 participants diagnosed with ASD (27% women and 73% men). Median age: 46 years old, with 52.38% participants under 50 years old and 47.62% aged 50 years or over.	The results highlight the importance of considering risk management and polypharmacy, mental, and neurological health problems. Prevention and treatment of specific conditions, such as epilepsy, kidney, and cardiovascular problems in elderly adults, are crucial to extending healthy life expectancy. Furthermore, identifying patterns of multimorbidity can provide insights into pathological aging and help personalize healthcare.	This study highlights the importance of ongoing healthcare and early geriatric assessment in people with ASD. Findings indicate potential associations between multimorbidity and the gut-brain axis, emphasizing the need for holistic strategies to improve quality of life.
Mogavero, Hsu(2020)^44^	Cross-sectional study (USA)	134 participants (46 diagnosed with ASD and 88 controls which diagnosis of ASD). Age range: 18–57 years old.	Many participants with ASD did not understand romantic relationships, although they had already shown interest or been in relationships (41.35%). Participants with ASD had lower percentages in the categories on how they learned to start relationships, with significant statistical differences between country (26.2%), colleagues (8.7%), media (30.4%), and social observation (50%). It was evident that the sexual orientations and minority gender identities showed increased prevalence among individuals with ASD.	Establishing sex education programs to reduce sexual anxiety and allow people with ASD to explore their sexuality with general social skills training and sex education. This education should teach courtship/dating behaviors and how to do so safely, especially if they are with potential partners, online, and be trained and taught how to recognize when someone is not interested and when someone is feeling bad about courtship.
Oh Mia et al.(2021)^18^	Intervention study (South Korea)	37 participants diagnosed with ASD (19 from the treatment group and 18 from the delayed treatment group). Average age: 23.5 years.	The Program for the Education and Enrichment of Relational Skills for Young Adults is an evidence-based intervention considered effective in improving relational skills in young adults with ASD. Implemented in young adults with ASD in South Korea, aiming to improve social and relational skills, it demonstrated effectiveness with a high completion rate of treatment (83.78%). After 4 months of participation, significant improvements were observed in the social skills, behavior and mental health of participants with ASD.	It highlights the reduction in symptoms of anxiety and depression, which resulted in significant improvements in the knowledge of social skills, involving the participation of parents as social coaches, with an emphasis on continuous practice. It is suggested that a comprehensive approach can positively influence the quality of life of these adults with ASD.
Pagni et al.(2020)^45^	Cross-sectional study (USA)	177 participants (95 diagnosed with ASD and 82 controls without diagnosed with ASD). Average age: 18–71 years.	Evidently, no significant differences were found between ASD and non-ASD groups in age, gender, and IQ. However, the participants without ASD had a statistically superior performance on the task compared to those with ASD; gender did not influence significantly.	It highlights the importance of understanding these variations in aging trajectories. This may be relevant for the development of specific and personalized therapies. The research highlights the need for future investigations into the underlying neural circuits and how these may influence aging trajectories, providing valuable insights to improve the quality of life of these adults with ASD.
Radhoe et al.(2023)^46^	Cross-sectional study (Netherlands)	720 participants (114 with diagnosis of ASD and 58 without diagnosed with ASD, in addition to a replication group with 261 with diagnosis of ASD and 287 controls without diagnosis ASD). Range age: 30–89 years old.	Based on variables related to aging and autism, of adults with autism, one of these research subgroups demonstrated greater vulnerability with more cognitive and psychological difficulties and lower quality of life. This finding suggests the need for specialized support and care for this specific group of adults with autism.	Not included.
Rocha et al.(2022)^47^	Cross-sectional observational study (USA)	108 participants (54 diagnosed with ASD; 54 controls no diagnosis of ASD). Age range: 18–58 years old.	Evidently, individuals with ASD may face specific challenges in the development of their sexuality, including a self-concept of less positive sex compared to individuals without ASD. Furthermore, a survey highlights the importance of adapting sexual education to meet the needs of people with ASD and highlights the need to better understand how sexual knowledge is related to sexual self-concept in this population.	A need arises for a model of psychosexual well-being that addresses the development of sexuality in adults with ASD. This sexually positive model will allow identified intrapersonal variables for the enhancement of a more in-depth examination of psychosexual well-being, which should assess whether some variables (sexual self-concept, sexual knowledge, and sexual feelings and attitudes) have a stronger influence on the sexual well-being of this population.
Roestorf, Howlin e Dermot(2022)^48^	Longitudinal study (UK)	440 participants (241 with diagnosis of ASD and 199 controls without diagnosis of ASD). Age range: 19–79 years old.	In this study, differences related to age, sex, and self-report versus third-party report in the characteristics of ASD in adults were analyzed. Adults with ASD presented higher scores in ASD characteristics and sensory sensitivity, with significant differences between sexes. Furthermore, discrepancies between self-report and third-party report, highlighting the complexity in understanding the characteristics of adults with ASD.	It highlights the importance of understanding the difficulties associated with aging and ASD. To improve quality of life, interventions focused on mental health, ongoing support and understanding specific factors such as interests and repetitive behaviors can be explored.
Schott et al.(2022)^49^	Retrospective observational cohort study. (USA)	622,468 participants (155,617 diagnosed with ASD and 466,851 controls no diagnosis of ASD). Age range: 18–64 years old.	Evidently, adults with autism were more likely to present psychiatric comorbidities, including depression, bipolar, anxiety, ADHD, OCD, schizophrenia, and other psychoses; however, they were less likely to present alcohol and drug abuse and bipolar disorder. They were also more likely to have physical health conditions such as Parkinson’s disease, endocrine disorders, epilepsy, nutritional conditions, and constipation. However, a lower chance exists of having disorders of the peripheral nervous system, paralysis cerebral, osteoarthritis, gout, and stroke, in that order.	Adults with ASD did not have a higher prevalence of some important health problems (e.g., cardiovascular problems, stroke, cancer, cardiovascular diseases), but they did have a higher probability of others (e.g., nutritional problems, epilepsy, central nervous system disorders).
Smith Da Walt et al.(2019)^50^	Longitudinal study (USA)	406 participants (adolescents and adults diagnosed with ASD, 72.4% men). Age range: ≥ 10 years (follow-up for 20 years). Average age: 21.4 years.	The predictors of mortality limited self-sufficiency in activities of daily living and deficiencies in social reciprocity. Approximately 6.4% of the sample died during the follow-up period. The average age at death was 39 years. Causes of death included chronicled conditions (such as cancer and seizures), accidents (such as choking on food and accidental poisoning), and health complications due to medication side effects.	Limited self-sufficiency responds to environmental influences, for example, employed adults with ASD are more likely to improve their skills because of expectations set by work. It is important to provide interventions designed to develop self-sufficiency in daily life for children, adolescents, and adults with ASD.
Torenvliet et al(2023)^51^	Longitudinal study (Netherlands)	240 participants (128 diagnosed with ASD and 112 controls which diagnosis ofASD). Age range: 24–85 years old.	Evidence against accelerated cognitive decline in adults with ASD exist. No significant differences were observed when comparing people with and without ASD in cognitive domains such as fluency, theory of mind, response speed, inhibition, and verbal memory. Learning among people with autism were shown to develop at a similar pace in both groups.	Not described.
Torenvliet et al.(2021)^52^	Longitudinal study (Germany)	176 participants (88 diagnosed with ASD and 88 controls without a diagnosis of ASD). Age range: 30–89 years old.	The group with ASD performed worse in several cognitive areas, including verbal fluency and the theory of the mind; however, performance was similar in visual memory. Furthermore, no age-related differences were shown in cognitive performance between adults with and without ASD.	Not described.
Torenvliet et al(2023)^53^	Longitudinal study (Germany)	254 participants (86 diagnosed with ASD 93 and 118 controls which diagnosis of ASD). Age range: 20–79 years old.	Comparisons exist between groups with ASD and controls revealed no significant differences in measures of prepotent response inhibition. However, when controlling response speed, adults with autism made more errors than adults without autism. Furthermore, a significant correlation exists between the hyperactivity/impulsivity scale and response variability in the group without autism.	Not described.
Tse et al.(2019)^54^	Cross-sectional study (England)	55 participants (28 diagnosed with ASD and 29 controls without a diagnosis of ASD). Age range:≥ 50 years.	The group with ASD and control group showed similar performance in verbal comprehension and perceptual reasoning; auditory, visual, and immediate and delayed memory, which suggests that having an autism diagnosis does not confer a risk factor for Alzheimer’s. People with ASD presented processing speed reduction. It is poorer performance on visual working memory tasks. It is suggested that the processing speed performance is closely related to levels of independence in daily functioning.	It is recommended that more research should be conducted on processing speed, as it is useful in promoting independent living in individuals with ASD. Therefore, with better knowledge of the cognitive functioning of elderly adults with ASD, the development of interventions and services for this population can be facilitated to meet their needs.
Uljarevic et al.(2020)^55^	Longitudinal study (Australia)	255 participants diagnosed with ASD (151 men and 104 women). Age:≥ 15 years. Average age: 43.75.	The study revealed that anxiety and depression are common in autistic adults throughout their lives, regardless of age. Additionally, women, people with more ASD symptoms, and individuals who live alone tend to have higher anxiety and depression scores.	It highlights the urgent need for adequate assessment and interventions throughout the lifespan to improve the quality of life of adults with ASD. However, the study does not detail specific intervention strategies.
Walsh et al.(2020)^56^	Cross-sectional study (USA)	85 participants diagnosed with ASD. Young people with an average age: 21.1 years Middle-aged adults: 53 years old.	Middle-aged men with ASD had higher scores on social cognition and showed reduced functional brain connectivity, especially in older men with ASD. Elderly adults with ASD had a higher incidence of epilepsy, but a lower incidence of depression/anxiety, regardless of intellectual disability.	This study highlights the importance of considering neurobiological factors and age groups when planning interventions and support for adults with ASD. A personalized approach, considering the specific needs of different age groups.
Walsh et al.(2022)^57^	Longitudinal study (USA)	50 participants (25 diagnosed with ASD and 25 controls no diagnosis of ASD). Age range: 40–70 years old.	A decline in the long-term visual memory of those with ASD, with the exception of shortterm, which is unchanged. Furthermore, hippocampal free water at baseline was found to be significantly correlated with long-term visual memory decline in the group with ASD, making this information important for a possible marker to predict age-related memory decline in the group with ASD.	Adults with ASD are at greater risk for accelerated memory decline, particularly long-term memory, as suggested by the higher incidence of early-onset dementia, the development of prognostic biomarkers will be an important development to inform early diagnosis and accurate treatment.
Yarar et al.(2022)^58^	Cross-sectional study (UK)	136 participants (79 diagnosed with ASD and 57 controls without diagnosed with ASD). Age range: 21–71 years Average age = 44.96 years.	Adults with ASD, both young and older, showed symptoms of autism without significant age-related differences. Both groups experienced mental health difficulties, with depression being the most influential factor in quality of life,surpassing IQ and severity of ASD symptoms. Age affected the quality of social life of older adults with autism, but had no impact on the control group.	Not described.
Zivrali Yarar et al.(2021)^59^	Cross-sectional study (UK)	97 participants (58 diagnosed with ASD and 39 controls without diagnosed with ASD). Age range: 18–50 years old.	No statistically significant differences presented at the intellectual level between groups. The group of young persons with ASD performed worse in theory fell: emotional perception, empathy and awareness of their emotions. A possible protective effect of age on theory of mind in people with ASD was identified. Elderly people with ASD showed higher levels of personal distress in tense social environments. The group with ASD showed higher levels of alexithymia when compared to the group of people with typical development.	Not described.
Zheng et al.(2021)^60^	Qualitative study (Australia)	15 participants diagnosed with ASD (53% women, and one nonbinary participant). Age range: 50–73 years old. Average age: 60.1 years.	Older adults with autism prefer everyday assistive technologies to manage the environment and increase accessibility. However, despite the assistance provided by technology in daily activities, gaps still exist in the support needs of this population, suggesting the importance of future research and guidelines to make the digital world more inclusive.	It highlights the need to explore specific categories of AT for cognition such as alerts and biofeedback to meet the emotional and cognitive needs of this population. Barriers to technology adoption such as cost, clear instructions, and early benefits are also discussed, highlighting the importance of overcoming them to promote effective AT use by older adults with ASD.

Notes: Attention Deficit Hyperactivity Disorder (ADHD), Autism Spectrum Disorder (ASD), Obsessive Compulsive Disorder (OCD), Intellectual Deficit Disorder (DID), Therapeutic Companion (AT), and Intelligence Quotient (QI).

 Eight articles (17.02%) included people with ASD associated with psychiatric comorbidities, such as intellectual development disorder (2.13%), anxiety and depression (6.38%), depression (2.13%), and others, such as attention deficit hyperactivity disorder (ADHD), obsessive compulsive disorder (OCD), and schizophrenia (2.13%). 

 The analyzed articles considered a diversity of approaches to diagnosis/confirmation of diagnosis of ASD: 1) standardized instruments, such as the Autism Diagnostic Observation Schedule (ADOS) and the Autism Diagnostic Interview-Revised (ADI-R), providing structured and objective assessments of ASD symptoms;^16,17^ 2) formal clinical assessment conducted by mental health professionals for direct observation of individuals’ behavior;^18^ 3) use of established diagnostic criteria, such as those from the Diagnostic and Statistical Manual of Mental Disorders (DSM-5) and the International Classification of Diseases (ICD-10), in different contexts to identify ASD;^19,20^ 4) combined analysis by multidisciplinary teams of specialists, conducting formal clinical diagnoses before inclusion and confirming the diagnosis with instruments such as the ADOS-2 and Autism Spectrum Quotient (AQ);^21^ 5) analysis of electronic health records extracted from medical institutions, providing additional information for diagnosis confirmation.^22,23^ The importance of confirming the diagnosis of ASD based on standardized instruments, such as those mentioned above, was also highlighted.^24^

### Cognitive aspects

 The microstructural integrity of the left dorsal premotor tract and supplementary motor area is directly associated with the severity of restrictive behaviors in adults with ASD.^35^ However, these adults demonstrated comparable performance to controls on skills such as verbal comprehension and perceptual reasoning. However, they face challenges, including reduced processing speed and difficulties in working and visual memory tasks.^54^

 Additionally, adults with ASD face difficulties in a range of areas, from empathy to sensory sensitivities and motor skills, highlighting the importance of ongoing support throughout life.^59^ One of the main findings was the variation in cognitive performance. While adults with autism demonstrated no significant differences from their neurotypical peers in domains such as fluency, theory of mind, response speed, or verbal memory, a decline in long-term visual memory was observed.^51^ This finding highlights the importance of early detection and targeted interventions to maintain cognitive health over time. 

 Paradoxically, age appears to exert a possible protective effect on the theory of mind in people with ASD, yet older adults with ASD face higher levels of personal distress in tense social environments.^59^ The relationship between aging and ASD is also evident in the reduction of brain connectivity, highlighting the importance of considering other factors that interfere with neurobiological aspects when planning interventions and support for adults with ASD.^21,56^

 Decreased mental flexibility in adults with ASD is associated with repetitive behaviors and variations in learning. The majority do not meet the criteria for "successful aging" because they present greater number of medical comorbidities and difficulties in accessing care–this aspect interferes with and implies greater difficulty in establishing and maintaining social relationships in everyday life.^36^

 Motor aspects are also affected, with deficits in manual dexterity, coordination, and strength, in addition to reduced functional connectivity between sensorimotor regions. The identification of these aspects indicates the potential for preventing accelerated functional decline through greater commitment, intervention with greater monitoring, and corroborating a better quality of life.^40^

 Although no significant differences in age, gender and Intelligence Quotient (IQ), adults with ASD exhibit a higher incidence of alexithymia are presented, they have greater difficulty recognizing and expressing emotions.^59^ Middle-aged men with ASD have higher scores on social cognition, while it was identified in studies that elderly adults with ASD face a higher incidence of epilepsy, but a lower incidence of depression and anxiety.^56^

 Adults diagnosed late with ASD show differential cognitive skill performance. Therefore, measures that improve executive function, such as practicing adequate physical activities and specialized medical monitoring, considering the individuality of each subject, are important to improve the quality of senescence for people with ASD.^19^

 Cognitive decline in adults with autism, along with possible accelerated neurophysiological aging, highlights the complexity of the interactions between aging and ASD.^51^ Thus, the delicate balance between cognitive and social characteristics throughout the lives of adults with ASD reveals the need for a more in-depth and personalized understanding to effectively address the challenges associated with this disorder during aging. 

### Aspects related to suffering/mental illness

 The relevance of psychiatric comorbidities in adults with ASD is an important element in adopting a functional approach, aiming to identify the underlying psychological suffering and adaptation strategies developed by these patients; furthermore, comorbidities seem to stand out for suffering in adulthood to the detriment of specific aspects of ASD.^32^ Data were found on the prevalence of mental health conditions, with anxiety and depression common in adults with autism regardless of age.^25^ Furthermore, the connection between cognitive functioning and other aspects of daily life, such as psychological distress in autistic individuals, presents a complexity that must be considered when personalizing healthcare for each adult with ASD.^25^

 Women with autism and people who live alone appear to be more susceptible to these conditions; therefore, adequate mental health resources essentially need to be available to this population.^22 ,38^ Furthermore, the results also indicate that adults with autism are more likely to present with other psychiatric conditions such as depression and anxiety, although they are less likely to abuse alcohol and drugs.^49^

 An alarming result was the high rate of suicidal ideation and suicide attempts among adults with autism.^34^ The research highlights the concern about suicidal ideation and self-injury, which have a higher rate in the autistic population, placing it as a risk group when compared to the general population.^34^ This highlights the importance of mental health care and providing appropriate support, particularly for those with bipolar and unipolar depression. Additionally, research has revealed that older adults with autism face difficulties coping with aging and often experience depression, anxiety, and sleep disturbances following the loss of loved ones.^23^ This emphasizes the need for long-term care emphasizing safety, medical monitoring, and therapy to address emotional challenges. 

 Specifically, in women with autism, they present higher psychological and somatic complaints, especially related to autism. Quality of life was found to be lower in individuals with clinical cutoff points indicative of depression.^41^

 The effect of comorbidities goes beyond psychological issues and influences communication and daily life skills. Constipation, epilepsy, and chronic kidney disease are the most frequent comorbidities associated with the severity of ASD.^42^ The importance of a functional approach is reiterated, highlighting that, in adulthood, the emphasis is on associated disorders and psychiatric comorbidities, rather than on the fundamental characteristics for the diagnosis of ASD.^32^

### Intervention strategies to improve quality of life

 Several strategies have been used to improve the quality of life of adults with ASD. Among these strategies, implementation of an evidence-based program designed to improve specific social and interpersonal skills in adolescents and young adults with ASD is particularly notable. Thus, the aim is to provide practical tools and strategies that not only reduce symptoms of anxiety and depression but also promote significant improvements in social skills. This program actively involves caregivers as social coaches, contributing to a more effective and widespread development of interpersonal skills in adult life.^18^ Furthermore, these interventions are essential and require personalized support, given that we understand the daily challenges faced by young people and adults with ASD; specifically for women with autism, this personalization is even more essential given their biological and social differences.^20,22^

 Within the scope of strategies to promote autonomy, individualized assessment and reduce polypharmacy,^23^ the need for specific approaches is highlighted. From this perspective, therapies focused on executive functions, physical activities, and specialized medical monitoring, for example, contributing to individual well-being, and understanding the variability of ASD throughout life favors the guidance of individualized approaches.^19,42^ Adaptive strategies such as emotional support and interventions focused on feelings of control were relevant when applied to different subgroups,^39^ and a mental health-centered approach and ongoing support are key to addressing the challenges associated with aging and autism.^46^ Thus, integration of these strategies offers promising avenues for addressing the needs of adults with ASD.^43^

 Adults with ASD demonstrate less vulnerability to cognitive decline when they are better educated, although they face elevated risks of memory decline, especially long-term.^61^ Mindfulness-based educational and stress reduction interventions benefit mental health-related quality of life, especially in women with ASD.^27^ Furthermore, professional activities, such as employment and higher education, are linked to positive outcomes and subjective well-being in adults with ASD. Career status at age 18 was identified as a solid indicator of professional outcomes in adult life, which emphasizes the importance of precocious interventions.^31^

 The relationship between greater social support and quality of life is also evident,^30^ highlighting the importance of solid support networks for adults with autism, especially as they age. The practice of mindfulness–techniques that direct full attention to present-moment experiences–can also help adults with autism learn to recognize and regulate their emotions, which can help alleviate the symptoms of psychiatric conditions comorbid with autism.^27^

 Furthermore, it is essential to develop a model of psycho-sexual well-being to better understand sexuality in this specific group.^47^ Adults with ASD reported lower levels of sexual desire and engagement in sexual behaviors. However, sexual satisfaction did not differ significantly compared to that in neurotypical people.^28^ This data reinforces the need for sexual education adapted to the specificities of people with ASD and the understanding of their unique sexual experiences. 

## DISCUSSION

 The growth/aging of people with ASD remains a little-explored topic in the literature, contrary to its description in children,^9^ which contributes to the persistence of obstacles related to the social and health demands of this population. Effective strategies to improve quality of life include early interventions focused on the integrity of brain areas related to restrictive behaviors,^35^ functional approaches considering psychiatric comorbidities, personalized adaptations,^32^ and interventions based on mindfulness for emotional regulation.^27^ Social support and support networks play a fundamental role in improving quality of life, especially during aging.^30^ These strategies have the potential to promote a better quality of life in adults with ASD. 

 In the mid-20th century, autism was included in the diagnosis of adult schizophrenia,^62^ because they had not yet had the individualization of the characteristics of a separate disorder with its particularities and assistance needs. Only in 2013, with the fifth edition of the *Diagnostic Manual of Mental Disorders*,^1^ the American Psychiatric Association recognized the unification of psychiatric conditions described in the literature as ASD. Thus, understanding how adolescents, adults, and elderly adults experience autism directly contributes to promoting quality of life for this population. 

 In 2019, the World Health Organization, in its eleventh edition of the *International Statistical Classification of Diseases and Related Health Problems*,^63^ unified ASD based on disorders reported in previous versions and subdivided it according to the presence or absence of intellectual disability and functional language impairment. From this perspective, given that ASD has specificities for each group of individuals, understanding such singularities is essential for individualized and assertive treatment, which helps in functional development, cognition and sociability,^64^ which are fundamental factors for maintaining the mental health of people with autism. 

 One clinical trial with young adults with ASD^18^ was successful in developing social skills and reducing anxiety and depressive symptoms among the intervention group based on a treatment focused on training interpersonal behavioral skills, with a satisfaction rate of 76.57% among study participants, with an average age of 23.5 years. This demonstrates how improving sociability directly influences the lives of adults with ASD, since even with communicative difficulties, interpersonal relationships are a part of the lives of people with autism.^65^

 Another study, covering this age group, found higher rates in variables related to happiness among a group of people aged 18–28 with ASD who had a job, possibly for the opportunity to contribute and establish social contact with individuals in their community.^31^ In addition to quality of life, social reciprocity in people with autism potentially influences their mortality, given that it is directly related to adaptive behavior and self-sufficiency.^50^ This point of social life must be introduced into the lives of people with autism to explore their cognitive potential, considering that aspects of verbal and visual memory of individuals with ASD may be similar to those of neurotypical people. However, it can manifest in different talents in each individual, with the potential for maturity through quality interpersonal relationships.^13^ Therefore, despite the specificities of communication between adults with ASD, the establishment of affective/social bonds is important for the well-being of this population. 

 Associated with these strategies, the recognition of depressive symptoms in individuals with ASD and provision of adequate support constitute a fundamental strategy to optimize the health of this population, especially because of the greater risk of suicidal ideation and self-harm among people with ASD, which are fundamental factors for negative outcomes of the condition. suffering psychiatric.^34^ In this sense, in addition to strengthening the family nucleus and support network of autistic adults, self-care practices, such as the implementation of leisure programs, physical activity, games and crafts, must be encouraged, in addition to practices that address spirituality and mindfulness.^66^ This speaks directly to the need of adults and elderly adults with ASD for emotional and cognitive self-knowledge, considering that they represent a risk group for psychiatric disorders such as anxiety and depression, especially with age, which can increase the risk of suicide.^28^

 In this sense, improved sleep quality is directly related to lower rates of suicidal ideation. Additionally, regular physical exercise is a protective factor against negative outcomes due to comorbidities. These practices have presented increasing benefits over the years, which can contribute to quality aging in people with ASD.^67^ Furthermore, a sense of belonging, artistic expression, and spirituality are effective for mental health care.^10^

 Art therapy, as a collective therapeutic strategy, contributes to the expression of feelings and thoughts, so that the inclusion of people with ASD in community-based services can contribute to the development of skills, social interaction, bonding, construction of perspectives of existence with and despite of the diagnosis(es).^68^ Together, such aspects of daily life must be addressed in the therapeutic plan for this population, considering the benefits to their mental health and quality of life. 

 In line with the social deficit in autism, the perception of topics such as aging and death is also affected by this disorder. Specifically among elderly adults, the danger of rupture and abandonment, especially during periods of loss of family members, illness, and the advent of old age, represent important risk factors for depression, which is often confused with premature aging, added to possible dietary symptoms, with an impact on greater vulnerability of this population group.^32^ However, daily difficulties can be managed with the use of currently available technologies that meet their needs, such as the use of the Global Positioning System (GPS), virtual reminders, messaging applications and headphones, capable of promoting the adaptation of elderly adults with ASD to daily life, providing autonomy by minimizing cognitive limitations that may impose themselves, such as the decline of visual memory and functionality.^57,60^ This does not require interventions for the prevention of emotional complexities that arise with the advent of aging, a phase in which family support and medical and psychological support are essential. 

 Questions about sex and sexuality were also addressed in some of the analyzed studies. Even though this field of research is small within ASD, people with autism clearly experience sexuality in a particular way, with unique experiences of the perception of sensuality, which is in dissonance with commonly guided sexual education, which is aimed at neurotypical individuals and rarely discusses self-perception.^47^ In addition to self-recognition of sex and sexuality, elucidating issues related to sexual vulnerability and interpretation of one’s own and other people’s body signs becomes a fundamental tool for the social development of people with autism, avoiding the infantilization of this population, and encouraging their autonomy.^69^

 Furthermore, addressing self-recognition of gender identity is also a benefit of sex education for individuals with ASD, considering that intimate issues can bring greater complexity to the social experience of these people, especially for transgender individuals, who often live with prejudice and the challenge of family acceptance, which can feed self-stigma.^70^ This phenomenon further aggravates the weaknesses in the process of growth and well-being of people with autism, which demands special attention from the social nucleus and healthcare team. 

 Considering the diversity of issues that permeate the adult experience of ASD, discussing strategies appropriate to individual reality is essential for improving quality of life in the process of growth and aging. 

 As this study is a literature review, limitations of the included studies may have impacted their results. In this sense, there is a low sampling rate in a considerable number of articles, often associated with the heterogeneity or diagnostic simplification of the individuals analyzed, in addition to the absence of control groups, which makes it difficult to generalize the results to a portion of the research. Other highlights are the cross-sectional approach in the methodology of some studies, limited information on changes over time, and the lack of studies conducted in developing countries. 

## CONCLUSION

 This integrative review reveals that adults with autism show strengths in verbal comprehension, perceptual reasoning, and theory of mind but face challenges such as reduced processing speed, sensory hypersensitivity, and difficulties in empathy and motor skills. They are more vulnerable to anxiety, depression, and suicidal ideation; however, they report similar sexual satisfaction as neurotypical individuals and lower substance abuse rates. 

 Effective strategies to enhance quality of life include evidence-based social skills programs, therapies for executive functions, physical activities, mindfulness interventions, and opportunities for education and employment. These approaches have contributed to the improvement of well-being and cognitive health over time. 

 This review emphasizes the importance of lifelong mental health support and tailored sex education for adults with autism. It also highlights the need for more research from developing countries on adult sexuality in autism to guide clinical practice and ensure comprehensive context-specific care throughout life. 
